# Communal farming, climate change adaptation and the media in Zimbabwe

**DOI:** 10.4102/jamba.v8i3.239

**Published:** 2016-03-04

**Authors:** Mthokozisi P. Ndhlovu, Thabani Mpofu

**Affiliations:** 1 Department of Journalism and Media Studies, National University of Science and Technology, Zimbabwe

## Abstract

Climate change is destroying Zimbabwean communal farmers’ agricultural activities – a source of living for most people. As communal farmers struggle to adapt, the media is expected to assume a fundamental theoretical role of educating and informing them about the appropriate adaptation techniques. Located in Umguza District in Matabeleland North Province, the study explored how communal farmers created meaning out of climate change media content and its influence on their agricultural practices from October 2014 to April 2015. In doing so, the study used the Two-Step Flow theory and Hall’s Encoding and Decoding Model. Entrenched in pragmatism, the study embedded quantitative techniques at different stages. Multistage sampling combining Simple Random Sampling (SRS), purposive and systematic sampling techniques was used to identify the 263 households for semi structured questionnaires, direct observations and in-depth interviews. The findings were analysed using Statistical Package for the Social Sciences (SPSS), thematic analysis and pattern matching. The results show that personal observations; print, broadcast and online media; and opinion leaders were the main sources of climate change information. The radio was the most used medium in communicating climate change adaptation though it was the second most accessed after mobile phones. Conservation Agriculture and planting of drought-resistant crops were some of the adaptation techniques communicated in the media. When interacting with media content, communal farmers create their own meaning influenced by their cultural values, resulting in some adopting, rejecting or modifying certain adaptation techniques. The study concludes that opinion leaders are fundamental in communal farmers’ interaction with media but their influence must not be overestimated.

## Introduction

The study, based in Umguza District in Matabeleland North Province, explored how communal farmers created meaning out of climate change adaptation media content and its influence on their farming practices from October 2014 to April 2015. Climate change is having a devastating impact on most agricultural activities in Zimbabwe. Climate change refers to changes in long-term weather patterns or averages, and this can be because of natural or external forces – attributed directly or indirectly to human activity (IPCC 2001; UNFCCC 1992). In Zimbabwe, climate change has largely manifested itself in declining rainfall patterns and increase in temperatures (Ministry of Environment and Natural Resources Management 2013). As communal farmers seem to struggle to make sense of and adapt to these catastrophic changes, the media is expected to assume a theoretical role of educating and informing them about the appropriate adaptation techniques. Adaptation in agricultural context refers to strategies farmers implement to maximise production regardless of changing climate patterns (Black *et al*. 2013). A broad definition of the media was adopted, encompassing micro-media – targeting a private and direct audience like social media- and macro-media – targeting a large audience ranging from mainstream radio and television to newspapers (Shelton 2013; Olesen 2007).

### Statement of the problem

Climate change is threatening the viability of agriculture – a source of livelihood for most Zimbabweans. The situation is even worse in Umguza District, as evidenced by a decrease in rainfall and an increase in temperatures, making adaptation fundamental if communal farmers are to improve their agricultural output (Ministry of Environment and Natural Resources Management 2013). Central to adaptation is the availability of information on the techniques communal farmers should implement – the role performed by the media. Studies conducted in Zimbabwe (Count-Evans 2013; Mare 2011) interrogated the media’s framing of climate change, neglecting the audiences. The study fills this gap through examining communal farmers’ interaction with climate change adaptation media content and its influence on their farming practices.

#### Background of the study

Climate change discourse has gained considerable debate in the media, policy and academic levels worldwide (Count-Evans 2013; Ministry of Environment and Natural Resources Management 2013; Pittock 2009). At international level, 195 countries have joined the United Nations Framework Convention on Climate Change (UNFCCC) (Castro 2014). This stimulated the researchers to explore how communal farmers created meaning out of media content of such a topical phenomenon and its influence on their farming practices. The Zimbabwean print, broadcasting and online media have reported on climate change adaptation.Newspapers like *Sunday News* have an agricultural section focusing on climate change issues. Television programmes like *Talking Farming* and *Murimi Wanhasi/Umlimi Wanamhla* broadcast by the Zimbabwe Broadcasting Corporation (ZBC) Television 1 also focus on climate change adaptation. Thus, communal farmers have access to different media content on climate change adaptation, but there is a need to understand how they access and create meaning out of that content and its influence on their farming practices.

In 2000, the government resettled communal farmers in parts of Umguza District. These farmers had no prior farming experience but were resettled in a semi-arid region susceptible to effects of climate change like declining rainfall and soaring temperatures, requiring them to adapt (Ministry of Environment and Natural Resources Management 2013).This also influenced the researchers to conduct a study examining how these communal farmers, some with less than 15 years farming experience, resettled in an area susceptible to the effects of climate change were interacting with mediated content on climate change adaptation and its influence on their farming methods.

#### Context of Umguza District

**FIGURE 1:**
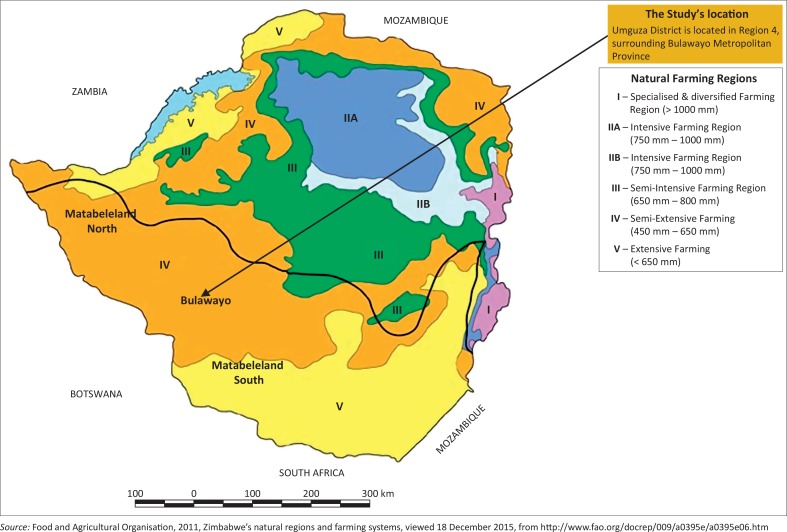
Zimbabwe’s agro-ecological regions.

Located in Matabeleland North Province, Umguza District closely surrounds Bulawayo Metropolitan Province, the country’s second largest city (see Figure 1). The District falls under Natural Agricultural Region 4, conducive for semi extensive farming, characterised by low levels of rainfall (Parliament of Zimbabwe 2011). Zimbabwe is divided into five agro-ecological zones, with best agricultural suitability being in Region 1 and least in Region 5. Apart from its location, Umguza District is impeccable for the study, as livestock and crop farming is a source of livelihood for most people in a region with high levels of poverty (Zimbabwe Election Support Network 2008).

#### An overview of the media in Zimbabwe’s Matabeleland region

Matabeleland region covers Bulawayo Metropolitan, Matabeleland South and Matabeleland North. The region accesses almost all macro-media reaching or based in Bulawayo including the *Chronicle*, a government-controlled publication and the *Southern Eye*, a private newspaper owned by Alpha Media Holdings (AMH). At the time of presenting and analysing data, the *Southern Eye* had shut down its print edition but continued publishing online and as a pull-out section in its sister daily newspaper, *News Day. The Zimbabwe Mail*, owned by the then Minister of Transport and Infrastructural Development, Dr Obert Mpofu, was also one of the publications based in Bulawayo before ceasing publication on 20 March 2015. This left the region with only one vibrant daily newspaper, the *Chronicle.* However, there are also three other daily publications based in Harare and accessible in the region – *The Herald,* a government-controlled publication, *News Day* owned by AMH and *Daily News* owned by Associated Newspapers of Zimbabwe (ANZ).

The weekly publications based in Bulawayo are *Umthunywa, B-Metro* and *Sunday News. Umthunywa* and *Sunday News,* the government-controlled publications, dedicate sections to farming in the Matabeleland region. Other weekly publications based outside Bulawayo, but accessible in the region are the *Sunday Mail,* a government-controlled publication and three privately owned weeklies, namely *The Zimbabwe Independent, Financial Gazette* and *The Standard*. Communal farmers can access news from short wave channels like Studio 7. They can also access television signal from Zimbabwe Broadcasting Corporation TV1 and radio stations like Radio Zimbabwe, Spot FM, Power FM, National FM, Star FM and ZiFM.

There are also various forms of micro-media accessible in the region. Because of its drought-prone location, Umguza District has several non governmental organisations (NGOs) operating to alleviate the effects of food shortages and in doing so they use newsletters to directly communicate with communal farmers. There are 16 international and local NGOs operating in the District, offering various agricultural-related services (Parliament of Zimbabwe 2011). Zimbabwe has witnessed a surge in the usage of Information Communication Technologies (ICT), translating to growth in the forms of media and adding social media in the category.Umguza District, because of its proximity to Bulawayo Metropolitan, enjoys good network coverage and other benefits brought by the surge in the usage of ICTs.

#### Objectives

The objectives of the study are to:

- Establish the sources of climate change adaptation information accessed by communal farmers in Umguza District.
- Find out the types of media used in communicating climate change adaptation.
- Explore the type of climate change adaptation information communal farmers receive from the media.
- Analyse communal farmers’ interaction with climate change adaptation media content.

#### Justification

Studies on the effects of climate change, such as those by Makadho (1996), Unganai (1996), Banda (2013) and the Ministry of Environment and Natural Resources Management (2013), came up with important findings, emphasising the need for climate change adaptation in developing countries. Banda (2013) warns that African countries must prioritise climate change adaptation if they are to increase their agricultural output. On the other hand, studies on media and climate change, such as that by Count-Evans (2013), focused on how the media communicates climate change with little regard to how farmers receive the information and its influence on their farming practices. The study filled this gap and the findings will assist policy makers, broadcast, print and online media content producers and development agencies to improve climate change communication and enhance the adaptation processes in Zimbabwe, as they will understand factors influencing communal farmers’ interaction with media content and its influence on their agricultural practices.

### Literature review

#### Evidence and impact of climate change in Zimbabwe

Climate change refers to changes in long-term weather patterns (climate) caused by natural or external forces – attributed directly or indirectly to human activity (IPCC 2001; UNFCCC 1992). There is documented scientific evidence indicating that Zimbabwe is experiencing adverse effects of climate change. Unganai (1996) contends that day temperatures soared by 0.8 °C from 1933 to 1993, whilst precipitation dropped by up to 10% during the same period. There have been severe dry spells, flooding, cyclones and heat waves linked to the impact of climate change (Ministry of Environment and Natural Resources Management 2013). As from 2000, tropical cyclones such as Cyclone Eline which made land fall in 2000, Cyclone Hudah and Cyclone Gloria have wreaked havoc, destroying buildings and agricultural produces. There have also been long dry spells with the most notable being in 1982, 1992 and 2002. The negative impact of climate change is worsened by the country’s dependence on agriculture, as it is an economic pillar.

#### Studies on media and climate change

Climate science and the media first came together in the coverage of climate change in the 1930s (Boykoff & Robert 2007). Since then, Peters (2012:2) contends that the relationship can be described as ‘distance, barrier, fence, oil and water and creative tension’. He adds that scientists and journalists are like strangers, unable to understand each other’s language.Despite these glaring differences, the media has a normative role of educating and informing its audience on climate change (Christians *et al*. 2009). Based on this, the study explored how Umguza District communal farmers generated meaning out of climate change adaptation media content created on conflicting principles and at times by journalists without an understanding of the phenomenon.

Since anthropological climate change (human induced) first emerged in mid-1980s, Moser (2010) maintains that the question on its communication has been topical. Luganda (2005) established that radio was the most used medium in communicating climate change in rural Africa, with Mare (2011) attributing this to the medium’s wider reach and compatibility with African rural settings. In Zimbabwe, a few studies have combined climate change and the media and most, if not all, focused on the media’s framing of the phenomenon, neglecting the audiences. Mare (2011) established that *Sunday Mail’s* coverage of climate change focused on negative stories quoting official sources whilst Count-Evans (2013) discovered that *The Standard* and *Sunday News* reported on climate change when there were events that could fit into the journalistic news values. These studies opened a gap for an audience-oriented study exploring their interaction with media content.

#### Theoretical framework

The study is grounded in Reception Theory and Hall’s (1978) Encoding and Decoding Model, Two-Step Flow theory and the Uses and Gratification Theory. Communication is a complex process with the audiences attaching meaning to media content either conforming or opposing the producer’s programmed meaning (Hall 1978). Fourie (2001:244) states that ‘Reception Theory investigates “readers” theoretical and empirical process of interpreting media texts’. Informed by this theory, the study investigated the meaning communal farmers attach to media content and its influence on their farming practices. Also fundamental in the process is Hall’s (1978) Encoding and Decoding Model.

Hall (1978) notes that media producers encode preferred reading in media content, but the audience decode and assign meaning to the content according to their different cultural experiences and background. The readings can be categorised as dominant, negotiated or oppositional (Hall 1978).Dominant reading is when the audiences accept the producer’s meaning, whilst in negotiated reading, they accept certain elements in media content and mix them with their own values and oppositional reading is when they totally reject the producer’s meaning and adopt the opposite. Hall (1978:52) adds that ‘if no “meaning” is taken, there can be no “consumption”. If the meaning is not articulated in practice, it has no effect’. As such, one of the three readings can be used to understand Umguza District communal farmer’s interaction with climate change adaptation media content.

The Uses and Gratification theory is useful in understanding the audiences’ interaction with media content. In the study, it was used to establish the reasons behind communal farmers’ usage of certain media products. Papacharissi and Rubin (2000) contend that according to the theory, audiences use certain media products to fulfil their needs. Using the theory allowed communal farmers to provide a description and reasons of their usage of certain media products.

Nisbet and Kotcher (2009) established that opinion leaders are necessary resources on climate change adaptation. Smith (2013) identified two forms of opinion leaders, namely formal opinion leaders comprising structural leaders elected to office and informal leaders exerting influence because of the knowledge of particular issues. This is in line with the Two-Step Flow theory, stating that media content moves in two stages. Steinberg (2007:266) notes that opinion leaders who are frequent media users obtain the information and ‘pass it along to those who are less exposed to media through informal and interpersonal communication’. It should be noted that whilst communicating the information, opinion leaders also add their interpretations and hence changing the meaning of the contents (Steinberg 2007).

## Research method and design

The study is rooted in pragmatism, combining positivism and interpretivism (Creswell & Plano-Clark 2011; Tashakkori & Teddie 2007). This offered the researchers the flexibility of mixing qualitative and quantitative techniques to answer the study’s research objectives demanding such answers. The study is a descriptive case study, and the embedded design strand of mixed methods was used to embed qualitative data within a traditional qualitative design (Creswell & Plano-Clark 2011). Both qualitative and quantitative data were concurrently collected, with the latter used for descriptive purposes. Three data-gathering techniques from across qualitative and quantitative techniques were employed, namely semi structured questionnaires, naturalistic direct observations and in-depth interviews. Qualitative and quantitative techniques were used to analyse data and to present findings, namely Statistical Package for the Social Sciences (SPSS), thematic analysis and pattern matching.

### Sampling

Multistage sampling combining SRS, purposive and systematic sampling was used to identify respondents for semi structured questionnaires and direct observations. The 11 respondents of structured in-depth interviews were purposively selected based on their positions in the District and their farming experiences. The researchers were taking notes during the interviews. Umguza District has 19 545 households with 87 518 people with an average of 4.5 individuals per household (Zimbabwe National Statistics Agency 2012). The District has 19 wards; 7 were purposively removed as they had a considerable number of commercial farmers who were not part of the study’s population. From the remaining 12 wards, wards 2 and 17 were selected, as they had the highest and lowest number of households, respectively. SRS with a margin error of ± 7.5, confidence level of 95% and an expected response rate of 95% was used to calculate the sample. Based on the SRS calculations, the researchers distributed 263 questionnaires supported by participant observations, with 169 being distributed in ward 2 with a population of 2775 households and 94 in ward 17 with a population of 191 households. Table 1 shows the distribution of questionnaires and observations across the two wards.

**TABLE 1:** Distribution of questionnaires and participant observations across Wards 2 and 17.

Ward	Sample
**Ward 2**	
Kensington	79
Springs Farm Village 1	30
Springs Farm Village 2	30
Heany Junction and Spring Farm Village 3	30
**Total**	**169**
**Ward 17**	
Majindane	31
Losikeyi	31
Maraposa	32
**Total**	**94**

**Grand Total**	**263**

## Ethical consideration

The study dealt with human subjects, hence bringing to the fore a host of ethical considerations the researchers observed. The principle of informed consent guided the researchers in the data-gathering process. Polit and Beck (2004:151) state that informed consent means that participants adequately inform the respondents about the nature of the research and they are capable of ‘comprehending the information and have the power of free choice, enabling them to consent voluntary to participate in the research or decline participation’. The respondents were also offered an option to opt out of the research whenever they felt uncomfortable. The researchers asked for approval from the Umguza Rural District Council to conduct the study. Because data was gathered at the household level, the researchers sought the participation of adults in all the sampled households. Again, all participants were informed of the nature of the research in terms of its scope and objectives and all the gathered information was treated with confidentiality.

### Dependability and trustworthiness

To achieve dependability and trustworthiness, the study used three different data-gathering techniques, namely semi structured questionnaires, in-depth interviews and naturalistic observation. In the study, internal validity was concerned with matching the research findings with the situation on the ground and was achieved through sampling and triangulation. The use of SRS with a confidence level of 95% and a margin of error ± 7.5 ensured that the sample is representative of the population, hence increasing internal validity.

## Results

### Overview of results

From the 263 distributed questionnaires, 236 were responded to, leaving only 28 that were not responded to, and all from Maraposa. The study findings were that personal observations and experiences, radio, broadcast, online media and opinion leaders were the main sources of climate change adaptation in the District. Communal farmers gained elementary understanding of climate change through personal observations and experiences of the phenomenon taking its toll on their agricultural activities. The media further developed their elementary understanding through educating and informing them about the changes. Also essential was the role of opinion leaders in influencing communal farmers’ interaction with climate change adaptation media content. The radio was the most used medium in the phenomenon, though it was the second most accessed after mobile phones, which were not fully used for adaptation purposes. Climate change adaptation information supported by various media content include planting of drought-resistant crops, conservation agriculture (CA), planting early-maturity crops and dry planting. When interacting with this content, communal farmers created their own meanings informed by their cultural values and lived experiences, resulting in them adopting, rejecting or modifying certain adaptation techniques.

## Discussion

### Sources of climate change adaptation information

**FIGURE 2:**
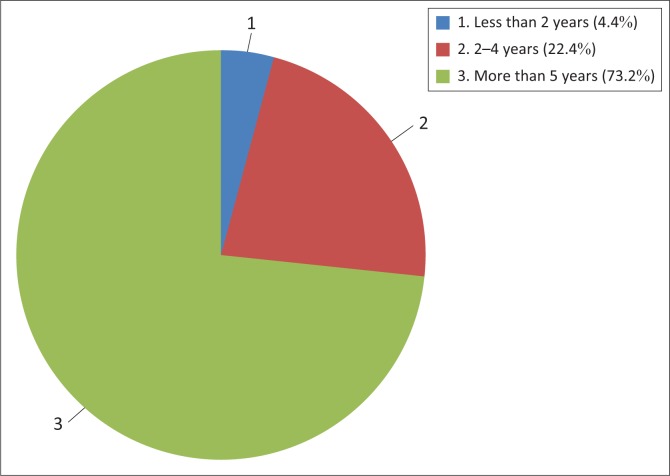
Communal farmers’ period of stay in Umguza District.

The study established that 99.1% of communal farmers knew about climate change and the need for adaptation, although to a varying degree and from various sources. Of those who participated in the study, 88.6% indicated that their rudimentary understanding was shaped by their observations and experiences of the phenomenon negatively affecting their agricultural activities. This was made possible by the years of farming experience they had congregated, thus allowing them to compare and contrast the District’s climate over the period. Figure 2 elaborates this.

As shown in Figure 2, 73.2% had stayed in the District for more than 5 years with the majority of them exceeding 15 years, allowing them to somewhat understand the District’s climatic patterns. As highlighted, most communal farmers in the District were resettled during the fast-track land reform programme that started in 2000, but those living in places like Kensington have been living there for more than 20 years. These communal farmers indicated that they observed that the District’s climatic patterns were changing. This was further captured by an elderly female respondent in Kensington who stated:

Long back around this time, our crops would be big, but this year as you can see it is now towards the end of December and we are still planting. The rains are nowhere to be seen and the temperatures are soaring. The climate is changing and this is clear for everyone to see. (Female, communal farmer, in her 60s)

Such sentiments summed up the experiences and observations of most communal farmers. To reinforce this, 87.3% said they observed that climate change was taking its toll on their crops, although the impact varied with the agricultural products and farming seasons. As such, 88.4% observed that their crops were dying because of shortage of rainfall and soaring temperatures. They added that this was now pronounced and increasing on a yearly basis, signalling the shifting climatic patterns. On the other hand, 18.4% mostly engaged in winter crop farming indicated that they had observed that unpredictable cold spells were destroying their crops, mostly horticulture.

Most communal farmers do not understand the technical nitty-gritties of climate change, but their observations and experiences captured all the necessary literature on the adverse impact of the phenomenon. To support this, Intergovernmental Panel on Climate Change (1992) agrees that climate change is a shift in the long-term weather patterns leading to unpredictable climatic patterns. Communal farmers’ observations and experiences all point to unpredictable climatic patterns that had been manifesting over a long period of time.

### Forms of media accessed by communal farmers

**FIGURE 3:**
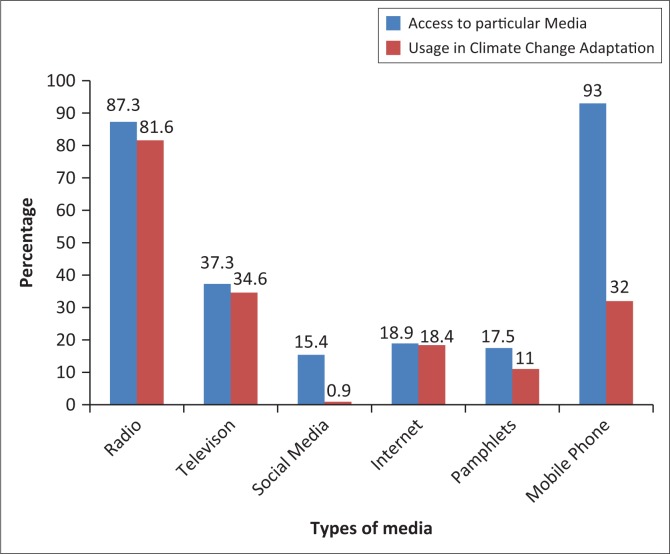
Types of media accessed by farmers in Umguza.

The study established that communal farmers had access to various forms of media and were using them at varying rates in efforts to adapt to climate change as illustrated in Figure 3.

As shown in Figure 3, the most accessed forms of media in the District are mobile phone (93%) and radio (87.3%), followed by television (37.3%). Internet (18.9%), pamphlets (17.5%) and social media (15.4%) are the least accessed. However, the study found out that access to a particular media does not necessarily translate to proportional use in climate change adaptation. Interestingly, the mobile phone at 93% is the most accessed, but at 32% usage for climate change it ranks third after radio (81.6%) and television (34.6%). The study discovered that micro-media were not being effectively used in communicating climate change adaptation. The study discovered that 15.4% were using social media platforms like WhatsApp and Facebook, but only 0.9% used the platforms for climate change adaptation, whilst 17.5% indicated reading pamphlets distributed by NGOs, but only 11% read pamphlets with climate change adaptation issues.

**FIGURE 4:**
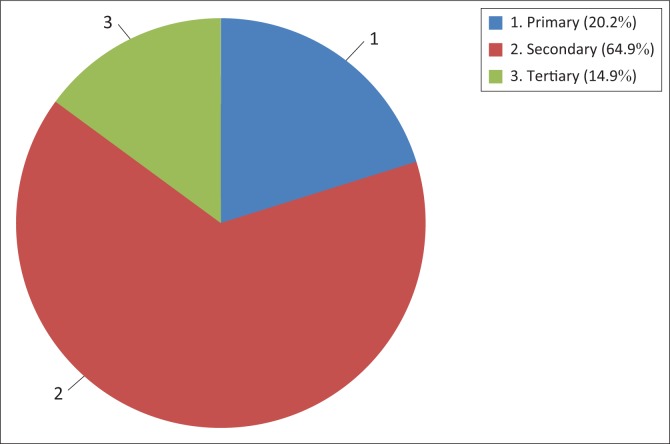
Communal farmers’ education levels.

Radio was the most used medium in communicating climate change adaptation. Of the participants, 87.3% had access to the medium and 81.6% used it for climate change adaptation. Some of the radio programmes on farming and climate change are *Ezabalimi, Ezezigodi, Inkundla Yabalimi, Green Matters* and *Indaba Zabalimi.* Communal farmers preferred radio to other forms of media mostly because of literacy levels and language preferences. Most communal farmers in the District speak *isiNdebele*, and radio channels such as Radio Zimbabwe and National FM use the language. The study established that there were some households with hardly a literate member, limiting their usage of certain forms of media such as newspapers where literacy levels are fundamental in consumption. Figure 4 explains this.

As demonstrated in Figure 4, 20.2% had their highest educated member doing or stopping at the primary level. At this level, pupils acquire elementary literacy and are able to write their names and other basic writing and reading techniques. This meant that most of the 20.2% were excluded from newspapers and other forms of media requiring advanced reading techniques. These findings support the studies by Luganda (2005) and Mare (2011) that established that radio is the most used medium in communicating climate change adaptation in rural Africa because of its wider reach and compatibility with the continent’s rural settings.

Television was the second most used information channel, as 37.3% had access to the medium and 34.6% indicated learning about climate change adaptation from the medium. However, the ownership of television in the District was skewed, as most people with access to the medium were from Kensington and surrounding areas, but the figures drastically dropped in areas like Springs Farm and ward 17. Most communal farmers who had access to television relied on programmes like *Talking Farming* and *Umlimi Wanamhla/Murimi Wanhasi* to obtain advice on the climate change adaptation issues they were facing.

The Uses and Gratification theory can be used to explain the selection of certain television and radio programmes in efforts to adapt to climate change. Papacharissi and Rubin (2000) argue that audiences select media content to fulfil their needs or wants. In this case, it can be argued that communal farmers consciously watched and listened to programmes like *Talking Farming* and *Murimi Wanhasi/Umlimi Wanamhla* to satisfy their specific desires, varying with each individual communal farmer based on the climate change farming challenges they were facing.

Third behind television in usage in climate change adaptation was the mobile phone. Although 93% had access to the gadget, only 32% used the medium in climate change adaptation. Mobile phones offer Internet services, which some communal farmers used to acquire information on climate change adaptation. One of the country’s mobile service providers, Econet Wireless Zimbabwe, offers farmers daily weather updates and farming tips to assist in their farming activities. In our study, 32% admitted reading about climate change and the need for adaptation on mobile phones. However, in most cases, communal farmers downplayed the role of mobile phones, as they themselves failed to understand that their actions were aimed at adapting to climate change. This misunderstood role was captured by a respondent who denied using mobile phones in climate change adaptation but said:

The Agritex officer stays far away from us, but if we are having problems like last year but one when our crops were not performing well we call him and he gives us advice through the phone. However, in some cases especially when the problem is affecting many villagers he comes. (Male, communal farmer, in his 40s)

### Entrenching understanding of climate change adaptation through opinion leaders

The study established that opinion leaders are highly respected by communal farmers in the District mostly because of their structural positions and achievements and they influence communal farmers’ interaction with media content. The interviewed opinion leaders were ward councillors and successful farmers. The influence of informal opinion leaders is widespread, an example is a farmer in Kensington who was only identified as Arab. He was identified by the Nyanga Research Station, a Department of Research and Specialist Services of the Ministry of Agriculture, Mechanisation and Irrigation Development, to partner them on a pilot project of growing various types of apples. The project catapulted Arab not only to be an informal opinion leader in Kensington but also in the District as the constituency’s Member of Parliament. Obert Mpofu who was also the Minister of Transport was the guest of honour in the official unveiling of the project. This influence of opinion leaders was summed up by the Village Head of Majindane, Sibanda who said:

When Agritex introduced *ugantshompo* [*Conservation Agriculture*], a few individuals were selected and trained on the techniques. These individuals became master farmers and started training other villagers the technique. (Sibanda [village of head of Majindane, Umguza District] pers. comm., 22 December 2014)

The findings show the importance of opinion leaders in influencing communal farmers’ interaction with media content, the same as Nisbet and Kotcher (2009:328) who established that opinion leaders are an essential ‘resource for collective action on climate change adaptation’. These findings can also be explained using the Two-Step Flow theory. However, unlike Nisbet and Kotcher (2009) and Steinberg (2007), the study established that the influence of opinion leaders is at times overestimated, as there are cases where they were over powered by observations and experiences of communal farmers. This was clearly visible in Majindane with the decline in the uptake of CA just a few years after being successfully introduced by Agritex with the support of opinion leaders. The researchers observed that many communal farmers were sceptical and had stopped using the technique despite encouragement from opinion leaders and the media, as they believed it was incompatible with their settings.

### Type of adaptation information communal farmers access from the media

The study established that communal farmers in the District accessed different types of climate change adaptation information from various media content. These techniques include planting of drought-resistant crops, early-maturity crops, dry planting, irrigation and CA.

### Communal farmers’ interaction with media content

#### Drought-resistant crops

**FIGURE 5:**
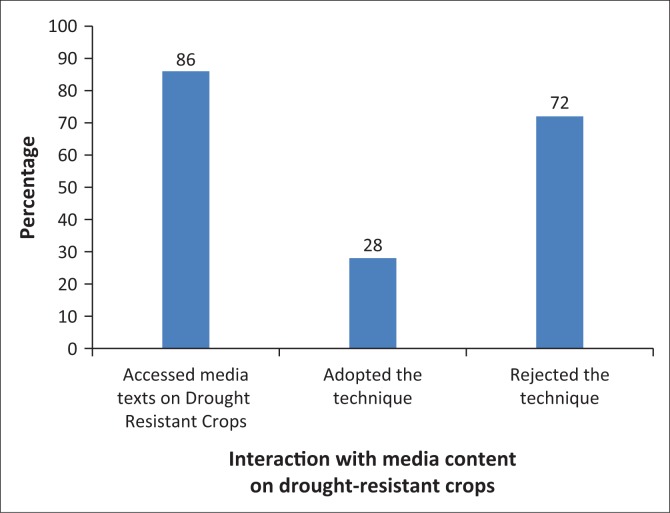
Farmers’ interaction with media content on drought-resistant crops.

There were several media material encouraging communal farmers to grow drought-resistant crops. These were in forms of media like radio, television and newspapers and pamphlets. Such media content encouraged communal farmers to plant crops like sorghum and millet, which are suitable for areas with little rainfall like Umguza District. Figure 5 provides more information.

As demonstrated in Figure 5, 86% accessed media content on drought-resistant crops, and of these households, 72% rejected the technique citing various issues key amongst them are the conflicting cultural backgrounds whilst the remaining 28% adopted the technique. This interaction can be explained using Hall’s (1978) Encoding and Decoding Model. Seventy-two percent of the communal farmers who accessed media content on drought-resistant crops totally rejected the encoded meaning, opting to adopt an oppositional meaning (Hall 1978). A female questionnaire respondent in Springs Farm Village 1 said:

There is no way my children can eat sorghum, as we came from an urban background. We were resettled here 10 years ago and we are not used to eating sorghum and millet. I will not grow the crops, unless if maybe I am forced. (Female, communal farmer, in her 50s)

This summarised the sentiments of most communal farmers who rejected growing drought-resistant crops regardless of accessing media content encouraging them to adopt the technique. Some communal farmers’ rejection of drought-resistant crops like sorghum was not only based on their dislike of eating the crop but also on the extra burden associated with growing the crop, as it attracted birds, thereby giving them an extra duty of chasing the birds from their fields.

### Conservation agriculture

Conservation agriculture (CA), popularly known as *gantshompo* or *Farming the God’s Way*, is one of the most popular climate change adaptation techniques in the District. Media content about this technique were available on radio, television, pamphlets especially from Agritex and NGOs and even newspapers. According to Food and Agricultural Organisation (2011:1), CA is a farming technique aimed at ‘managing agro-ecosystems for improved and sustained productivity, increased profits and food security while preserving and enhancing the resource base and the environment’. Twomlow and Hove (2006) explain the technique:

In southern Zimbabwe it is recommended that the planting basins be dug each year from early August through October in the same positions. The recommended dimension of each basin is 15 cm (length) × 15 cm (width) × 15 cm (depth) and the basins are spaced at 90 cm × 60 cm. Available soil fertility amendments (organic and/or inorganic fertilizers) are then added to each basin which is then lightly covered with soil in September/October. Rain water is collected in the basins during the early season rainfall events (October and November). Planting follows in November or December after the basins have captured rain water at least once. (p. 1)

**FIGURE 6:**
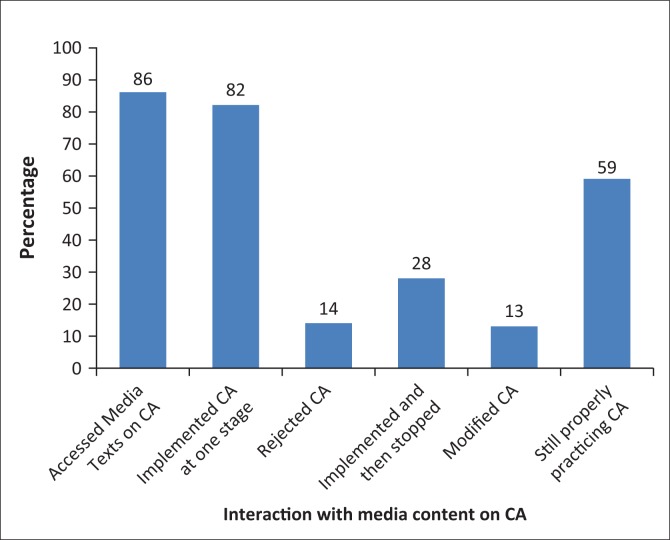
Farmers’ interaction with media content on conversation agriculture.

The study established that communal farmers interacted with media content of CA in different ways. Figure 6 provides more details.

Figure 6 illustrates that 86% indicated accessing media content on CA and 82% admitted implementing the technique at one stage. Of the remainder of those who accessed media content, 14% rejected the technique because of various reasons, chief amongst them being its labour-intensive nature. It must be highlighted that amongst those who implemented the technique at one stage, 28% stopped using it, saying it was not compatible with their land whilst others complained about its labour-intensive nature. Also among those who implemented the technique at one stage, 13% modified the technique to fit their circumstances. The remaining 59% are still practicing the technique and are trying by all means to adhere to the methods espoused by media content.

This interaction with media content on CA can be best explained using the Encoding and Decoding Model. Despite media content encouraging them to adopt the technique, 14% of communal farmers who accessed the content adopted oppositional reading and rejected the technique. Most of these farmers complained about the labour intensiveness of the technique. A total of 59% of the farmers adopted dominant reading and implemented the technique, trying by all means to follow the method as stipulated by media content. However, 13% of the communal farmers adopted preferred reading mixed media content with their own practices. An old-aged female questionnaire respondent in Kensington explained:

You see some of us are very old and we do not have the energy to use the technique, but we believe that it is productive. As such, we are using our own *gantshompo* (CA) where we dig very small holes and do not even put manure. These holes capture water and as such we do get a better harvest although the technique we are using is not the *gantshompo* (CA) that most people use. (Female, communal farmer, in her 80s)

### Challenges faced by communal farmers when using the media in efforts to adapt to climate change

In interacting with media content in efforts to adapt to climate change, communal farmers face numerous challenges. The challenges are two fold: those originating from the composition of media content and others emanating from the structural problems governing the media content’s distribution. These challenges disturb communal farmers’ interaction with media content and the process of meaning making.

### Unreliable media information on climate change

Communal farmers expressed concerns on depending on media information in efforts to adapt to climate change, saying some information was unreliable. To support this, 62% said they did not completely trust the media content’s weather and climate predictions, as there were instances where they misinformed them, making it difficult for them to plant according to the media’s predictions. To support this, one of the communal farmers added:

At times, the radio and television tell us wrong information on when it will start raining. However, the rains do not necessarily start as according to the predictions, resulting in our crops drying up due to shortage of rain. (Male, communal farmer, in his 40s)

Given the unreliability of media texts weather predictions, communal farmers now resort to mixing the media predictions with their farming experiences before planting to avoid their crops drying up because of the shortage of rain and soaring temperatures. It must be noted that communal farmers, because of the lengthy period they have lived in the District, have acquired an understanding of the area’s climatic patterns; hence, mixing their experiences with media texts predictions reduces the chances of making wrong predictions.

### Language used in communicating climate change

Another challenge faced by communal farmers when using the media in efforts to adapt to climate change is rooted in language misunderstanding. Most communal farmers in Umguza District speak isiNdebele; however, some media texts use Shona and English. An example of such a programme is *Talking Farming,* where the farming experts at times will be only speaking in Shona and English, making it difficult for some communal farmers who do not understand the two languages to comprehend what they would be talking about. Somampisi Sibanda of Kensington adds:

We are neglected, as some programmes like *Murimi Wanhasi* use Shona and we do not understand Shona. Fine, we might understand a few things, but we certainly miss other important information. (Somampisi Sibanda [communal farmer and a leader in Kensington, Umguza District] pers. comm., 25 January 2015)

Some communal farmers expressed concerns over the media use of jargons, which they said made it difficult for them to adapt to the changing climatic patterns, as in most cases they fail to understand the meaning of those words. This supports Peters (2012) observation that scientists and journalists are like strangers, unable to understand each other’s language whilst Hartz and Chappell (1997:22) add that ‘many otherwise well-educated writers and reporters have never taken the time to become familiar with the culture of science, its language and its methods’. As such, this relationship between science and journalism negatively affects the way communal farmers interact with media texts in efforts to adapt to climate change, as they will also fail to understand the meaning of certain climate change jargons in some media texts. The literacy levels of communal farmers in the District highlighted earlier further make it difficult for them to grasp these jargons, which even the journalists also fail to understand at times.

### Media focus on unaffordable and irrelevant techniques

Climate change adaptation media content contains some techniques that are inapplicable to Umguza District or too expensive for communal farmers to implement. This is a big challenge to communal farmers. In such a case, communal farmers analyse various adaptation techniques in media and select those that are applicable to their settings. To support this, communal farmers noted that some of the adaptation techniques espoused by the media texts were not designed for Umguza District. One of the communal farmers added ‘we listen to all the adaptation techniques in the media and after that we pick the techniques that we feel might assist us and leave out the rest’ (Male, communal farmer, in his 50s). In some cases, communal farmers face financial challenges as they do not have the required money to implement certain adaptation techniques. For example, building a proper greenhouse is expensive for most, if not all, communal farmers in Umguza District. Despite such financial challenges, some communal farmers built their own effective homemade greenhouses using locally sourced materials.

## Limitations of the study

The major limitation was the study’s sampling, as it used a big sampling margin of ± 7, 5. The study also failed to obtain responses from 28 questionnaires distributed in Maraposa.

### Recommendations

Producers of broadcast, print and online climate change adaptation media content must take advantage of ICTs to package adaptation information. Most communal farmers in the District have access to mobile phones, but are not using them for climate change adaptation. There is need for producers of media content on climate change adaptation to understand the cultural values and lived experiences of communal farmers to maximise chances of adoption of the communicated media content. The study recommends that producers of climate change adaptation content must incorporate opinion leaders in their strategies as they have a huge influence on how communal farmers interact with certain adaptation techniques. However, the influence of opinion leaders should not be overestimated as it can also be rejected if it does not incorporate communal farmers’ experiences and observations.

## Conclusion

The study concludes that availability of media platforms does not necessarily translate into their usage in climate change adaptation. The study has shown that whilst the mobile phone is the most common medium, it is not widely used for climate change adaptation. The study also concludes that whilst social media, Internet and other forms of micro-media have revolutionised communication worldwide, communal farmers in Umguza District have not adopted these as major platforms for communicating climate change. The study also concludes that communal farmers actively interact with media on climate change adaptation and reject any adaptation technique failing to incorporate their cultural values and experiences. It is also the study’s conclusion that opinion leaders are fundamental in communal farmers’ interaction with media content but their influence must not be overestimated.
